# Identification of proteins involved in the functioning of *Riftia pachyptila *symbiosis by Subtractive Suppression Hybridization

**DOI:** 10.1186/1471-2164-8-337

**Published:** 2007-09-24

**Authors:** Sophie Sanchez, Stéphane Hourdez, François H Lallier

**Affiliations:** 1 Equipe Ecophysiologie: Adaptation et Evolution Moléculaires, UMR 7144 CNRS UPMC, Station Biologique, Place Georges Teissier, BP 74, 29682 Roscoff Cedex, France

## Abstract

**Background:**

Since its discovery around deep sea hydrothermal vents of the Galapagos Rift about 30 years ago, the chemoautotrophic symbiosis between the vestimentiferan tubeworm *Riftia pachyptila *and its symbiotic sulfide-oxidizing γ-proteobacteria has been extensively studied. However, studies on the tubeworm host were essentially targeted, biochemical approaches. We decided to use a global molecular approach to identify new proteins involved in metabolite exchanges and assimilation by the host. We used a Subtractive Suppression Hybridization approach (SSH) in an unusual way, by comparing pairs of tissues from a single individual. We chose to identify the sequences preferentially expressed in the branchial plume tissue (the only organ in contact with the sea water) and in the trophosome (the organ housing the symbiotic bacteria) using the body wall as a reference tissue because it is supposedly not involved in metabolite exchanges in this species.

**Results:**

We produced four cDNA libraries: i) body wall-subtracted branchial plume library (BR-BW), ii) and its reverse library, branchial plume-subtracted body wall library (BW-BR), iii) body wall-subtracted trophosome library (TR-BW), iv) and its reverse library, trophosome-subtracted body wall library (BW-TR). For each library, we sequenced about 200 clones resulting in 45 different sequences on average in each library (58 and 59 cDNAs for BR-BW and TR-BW libraries respectively). Overall, half of the contigs matched records found in the databases with good E-values. After quantitative PCR analysis, it resulted that 16S, Major Vault Protein, carbonic anhydrase (RpCAbr), cathepsin and chitinase precursor transcripts were highly represented in the branchial plume tissue compared to the trophosome and the body wall tissues, whereas carbonic anhydrase (RpCAtr), myohemerythrin, a putative T-Cell receptor and one non identified transcript were highly specific of the trophosome tissue.

**Conclusion:**

Quantitative PCR analyses were congruent with our libraries results thereby confirming the existence of tissue-specific transcripts identified by SSH. We focused our study on the transcripts we identified as the most interesting ones based on the BLAST results. Some of the keys to understanding metabolite exchanges may remain in the sequences we could not identify (hypothetical proteins and no similarity found). These sequences will have to be better studied by a longer -or complete- sequencing to check their identity, and then by verifying the expression level of the transcripts in different parts of the worm.

## Background

The vestimentiferan annelid *Riftia pachyptila *lives around hydrothermal vents on the East Pacific Rise at 2600 meters-depth. These giant tubeworms form dense aggregations and constitute a major component of the biomass in these deep-sea oases of life that rely on chemosynthetic primary production [1]. Adult vestimentiferans lack a mouth, gut and anus [2]. Instead, they possess a specialized tissue, called trophosome, that contains symbiotic bacteria. This symbiosis with sulfide-oxidizing bacteria provides all the host's nutrition and is therefore obligatory [3]. Their larvae however, possess a digestive tract [4], and are devoid of symbiotic bacteria which they acquire from the environment. The acquisition of bacteria occurs through the skin, and the trophosome is established from mesodermal tissue. Then, apoptosis of infected cells in the host epidermis occurs at the end of the colonization process [5].

Several studies focused on the functioning of this symbiosis. Previous biochemical and enzymatic studies addressed the uptake of hydrogen sulfide [6,7] and the transport of both oxygen and hydrogen sulfide by the giant extracellular hemoglobins [8-10]. The diffusion of carbon dioxide through the branchial plume [11] and its subsequent conversion into bicarbonate through the activity of carbonic anhydrase [12,13] were also demonstrated. More recently, molecular techniques were used to better understand some aspects of the exchange mechanisms in the branchial plume and the trophosome, such as the existence of a carbonic anhydrase transcript[14]. The sequencing of the whole genome of the symbiont of *Riftia pachyptila *is currently under progress (Horst Felbeck, personal communication) and a proteomics approach has been carried out on the symbiont [15] revealing previously unsuspected carbon fixation pathways. However, no global genomic work on the host has been published to date.

Identification of differentially-expressed transcripts (i.e. transcripts which differ in abundance between samples being compared) has been conducted for the last ten years on symbiotic interactions between rhizobia and legumes (for review see [16]) thanks to improved molecular approaches such as Subtractive Suppression Hybridization (SSH), for example. Morel and coworkers [17] constructed cDNA libraries by a SSH procedure and performed hybridizations on arrays between two compartments of the fungus *Paxillus involutus *living in symbiosis with the plant *Betula pendula*. These methods successfully identified differentially-expressed sequences in this ectomycorrhizal symbiosis, suggesting differences in metabolism between the two studied compartments [17]. SSH appears to be a quick and efficient method to rapidly obtain many specific sequences. It is a powerful method to enrich samples for differentially expressed transcripts by combining steps of suppression and normalization prior to differential screening, and this starting from very little material.

A transcriptome analysis of a marine cnidarian-dinoflagellate symbiosis using microarrays to compare aposymbiotic and symbiotic stages of the host *Anthopleura elegantissima *revealed the existence of key genes involved in the maintenance of the symbiosis [18].

In *Riftia pachyptila*, aposymbiotic larvae/post-larvae are very small (less than 100 μm) and very difficult to obtain. In addition, the host cannot be kept alive without its symbionts. Therefore, comparison between aposymbiotic and symbiotic states in *R. pachyptila *cannot be considered at present. Previous studies on the host were only targeted molecular studies and no global molecular analysis has been carried out on the host *Riftia pachyptila *to date. The aim of the present study was to identify host transcripts that could be involved in metabolite exchanges in the branchial plume on the one hand, and in metabolite exchanges with the symbionts in the trophosome. We postulated that these specific protein-coding genes should be preferentially expressed in these two tissues that are directly involved in the symbiotic way of life. Instead of the usual application of SSH that compares the same tissue in two physiological states, we compared pairs of tissues from a single individual. The subtracted libraries obtained should therefore be enriched in specific sequences compared to a classical library without any subtraction procedure. In theory, identical sequences between key tissue and the reference tissue (housekeeping genes sequences in particular) should be eliminated by the subtractive suppression hybridization. Only tissue-specific sequences should be recovered in each library. We also maximized the chances of obtaining new sequences using the normalization procedure which increases the amount of rare transcripts during the SSH procedure.

## Results

### General results of sequencing

Global results including the total number of obtained sequences, contigs, singletons, and redundancy rates are given in Table 1 for all the libraries (the body wall-subtracted branchial plume library (BR-BW), the branchial plume-subtracted body wall library (BW-BR), the body wall-subtracted trophosome library (TR-BW) and the trophosome-subtracted body wall library (BW-TR)). The redundancy rates of the libraries range from 80.5 to 95.6 %. This indicates that additional sequencing should bring few or no new sequences. The sequences obtained were assembled into 58, 45, 59 and 17 different sequences (each putatively representing one cDNA) respectively for each library. Of those, 38, 17, 36 and 6 appeared as singletons, respectively.

**Table 1:** Overall statistics based on the analysis of each library

	BR-BW	BW-BR	TR-BW	BW-TR
Number of fragments sequenced	202	165	185	137
Number of cDNAs (contigs)	58	45	59	17
Number of contigs formed by one sequence (singletons)	38	17	36	6
Redundancy rate	81.2	89.7	80.5	95.6

**Figure 1:**
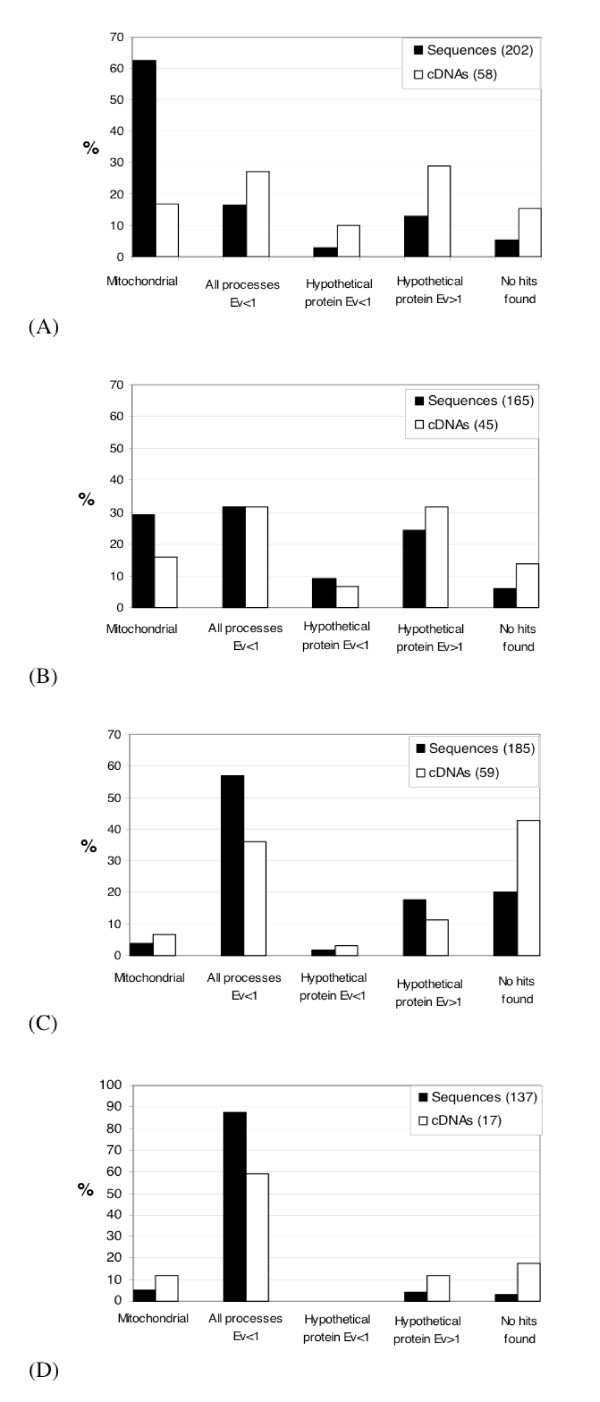
Proportion of sequences and contigs split into 5 main categories (mitochondrial, all processes sequences (E-value<1), hypothetical sequences (E-value<1), hypothetical sequences (E-value>1) and no similarity found). (A) Results for the BR-BW cDNA library. (B) Results for the BW-BR cDNA library. (C) Results for the TR-BW cDNA library. (D) Results for the BW-TR cDNA library.

Figure 1 shows the proportion of sequences with homology or not in the GenBank protein database for the four libraries: BR-BW (Fig. 1A), BW-BR (Fig. 1B), TR-BW (Fig. 1C) and BW-TR (Fig. 1D). These sequences are split into different categories: 1) mitochondrial sequences, 2) all processes sequences (with E-value < 1), 3) hypothetical sequences (with E-value < 1), 4) hypothetical sequences (with E-value > 1) and 5) no similarity found. If we consider that the two last categories cannot improve our knowledge, it remains that the proportions of cDNAs which matched with good homologies scores in GenBank database are 54.2 % in the BR-BW, 54.6 % in the BW-BR, 45.9 % in the TR-BW and 70.6 % in the BW-TR libraries. The choice of E-value = 1 as the threshold to assess the degree of similarity to protein sequences in GenBank database is arbitrary: we are well aware that E-values of 10^-3 ^to 10^-5 ^are usually chosen but, given that the sequences we obtained are relatively short and that there is little molecular data on vestimentiferans or annelids in general, we decided to use this high threshold. For example some cytochrome c sequences (BRbwC9) showed high E-values (0.11, see Table 2) although sequence identity reached 81% based on a 16 amino acid alignment.

**Table 2:** List of contigs with best E-values obtained for the BR-BW library sequences

Contig (BRbwC)	Number of sequences per contig	Putative identification on Blastx	E-value	GenBank accession number*
***Mitochondrial sequences***				

1	78	rRNA 16S large subunit 2390–2967	0	AY741662.1
2	32	rRNA 16S large subunit 2962–3499	0	AY741662.1
3	7	cytochrome c oxidase (ccox) subunit I 12–729	0	AAU20751.1
4	2	ccox subunit I 735–1548	0	AAU20751.1
5	1	ccox subunit Vb	0.084	EF648457
6	1	ccox subunit VIc	6.10^-13^	EF648477
7	1	ccox *Proteus vulgaris*	0.19	EF648458
8	2	ccox subunit Va	4.10^-28^	EF648459
9	1	ccox subunit Vb	0.11	EF648460

***All processes sequences (E-value<1)***				

10	2	ATP F0 c subunit	3.10^-16^	EF648461
11	1	ATP F1 β subunit	4.10^-93^	EF648462
12	11	carbonic anhydrase	3.10^-12^	EF490380
13	4	Major Vault Protein	6.10^-20^	EF648463
14	1	chitinase precursor	3.10^-8^	EF648464
15	*3*	cathepsine L-like	0.004	EF648465
16	2	BTG1 protein	2.10^-21^	EF648466
17	1	α-tubuline	3.10^-5^	EF648467
18	1	hydroxylamine reductase	3.10^-20^	EF648468
19	1	transcription repair coupling factor	0.67	EF648469
20	1	transcriptional regulator	0.66	EF648470
21	1	valosin containing protein	0.013	EF648471
22	1	Rab5 GDP/GTP exchange factor	0.65	EF648472
23	1	serine protease	0.56	EF648473

***Hypothetical protein (E-value<1)***				

24	1	super cystein-rich protein	5.10^-8^	EF648474
25	1	hypothetical protein	2.10^-22^	EF648475
26	1	hypothetical protein	2.10^-5^	EF648476

***Unknown sequences (E-value>1 or No Hits Found)***				

27 to 58	GenBank accession numbers EF648478 to EF648509			

### Specific sequences obtained from each subtracted library

#### Subtracted branchial plume library (BR-BW)

Fig. 1A shows a strikingly high proportion of mitochondrial sequences (62.4%) compared to what is observed in the three other libraries. Table 2 shows the sequences with the best E-values for the sequences obtained from the branchial plume library. First of all, among the most redundant clones, we found a 16S ribosomal mitochondrion sequence, which appeared highly redundant (contigs 1 and 2, corresponding to two fragments of mt16S, contain 78 and 32 sequences, respectively). High homologies scores were obtained for several contigs, in particular contigs 12 (carbonic anhydrase), 13 (Major Vault Protein), 14 (chitinase precursor), 15 (cathepsin L-like), 16 (BTG1 protein), 17 (α-tubuline), 18 (hydroxylamine reductase), and 24 (super cystein rich protein). The carbonic anhydrase cDNA obtained in the branchial plume (hereafter referred to as RpCAbr) was different from the one already sequenced from a trophosome cDNA sample [12] (hereafter referred to as RpCAtr). The RpCAbr full-length sequence (GenBank:EF490380, [19]) is only 66% identical in amino acids to RpCAtr (GenBank:AJ439711, [12]).

#### Subtracted body wall library (BW-BR)

The sequencing of the reciprocal library (i.e. BW-BR) revealed only 3 cDNAs in common with the BR-BW library: the two sections of the mitochondrial rRNA 16S large subunit (although they form a smaller proportion of the sequences), and the branchial carbonic anhydrase (RpCAbr) cDNAs. Although it was not the main target, this library yielded interesting sequences involved in the formation of the tube and therefore expected to be specific of the body-wall (Table 3), including a *Riftia pachyptila *exoskeleton β-chitin-binding transcript (contig 9) almost identical (2 differences over 74 amino acids aligned) to the one sequenced by Chamoy and coworkers [20]. We also obtained a different exoskeleton β-chitin-binding transcript (contig 10) with a highest homology score with the previously sequenced one [20]. Surprisingly, contig 11 showed a high homology with galaxin, a protein present in the calcified exoskeleton of the coral *Galaxea fascicularis*. Two transcripts coding for respiratory proteins were also found in this library: a new extracellular hemoglobin linker (contig 14) [21] which matches with *Sabella spallanzanii *linker chain sequence, and an intracellular globin (contig 15).

**Table 3:** List of contigs with best E-values obtained for the BW-BR library sequences

Contig (BWbrC)	Number of sequences per contig	Putative identification on BlastX	E-value	GenBank accession number*
***Mitochondrial sequences***				

1	10	rRNA 16S large subunit 2390–2967	0	AY741662.1
2	10	rRNA 16S large subunit 2962–3499	0	AY741662.1
3	1	cytochrome c oxidase (ccox) subunit I 293–471	0	AAU20751.1
4	12	ccox subunit I *Littorina*	2.10^-77^	EF648510
5	9	ccox subunit I *Paralvinella*	3.10^-172^	EF648511
6	2	ccox subunit II	0	AAU20752.1
7	4	ccox subunit IV *Urechis*	4.10^-50^	EF648512

***All processes sequences (E-value<1)***				

8	1	carbonic anhydrase	3.10^-12^	EF490380
9	9	exoskeleton β-chitin-specific binding (3)	1.10^-43^	EU131642
10	9	exoskeleton β-chitin-specific binding (2)	1.10^-7^	EF648513
11	6	galaxin	2.10^-14^	EF648514
12	7	myosin regulatory light chain 102–576	0	AF173680
13	4	myosin regulatory light chain *Eisenia*	3.10^-12^	EF648515
14	2	extracellular hemoglobin linker	0.39	AM000033
15	2	intracellular globin	2.10^-9^	EF648516
16	6	troponin C	2.10^-13^	EF648517
17	2	similar to fraser syndrome 1 isoform 1	0.007	EF648518
18	1	sugar transporter *Arabidopsis*	0.79	EF648519
19	1	putative integrin	0.14	EF648520

***Hypothetical protein (E-value<1)***				

20	13	hypothetical protein	0.1	EF648521
21	1	hypothetical protein	0.62	EF648522
22	1	unamed	0.13	EF648523
23	1	hypothetical protein	0.047	EF648524
24	1	hypothetical protein	0.002	EF648525

***Unknown sequences (E-value>1 or No Hits Found)***				

25 to 45	GenBank accession numbers EF648526 to EF648546			

#### Subtracted trophosome library (TR-BW)

Sequences obtained from this library were compared to the unpublished genomic sequences of the symbiont (with Horst Felbeck permission) in order to verify that they were host-specific, and not a contamination from the symbionts that are contained in this tissue. The trophosome library yielded much less identifiable sequences (Table 4). Noticeably, among the identifiable sequences, we recovered the previously sequenced carbonic anhydrase transcript (RpCAtr) [12], and transcripts coding for a large number of globin chains (contigs 8–15), for a hemoglobin linker (contig 16), and for a myohemerythrin (contig 17). Among the cDNAs with a significant blast value (all processes sequences (E-value<1)), more than 56 % are respiratory pigment protein transcripts. These latter are partial fragments of the already known A1, A2, B1, B2 chains of the giant extracellular hemoglobin of *Riftia pachyptila*, and a probably new A2 (contig 12) and B1 (contig 14) chains. The extracellular hemoglobin linker identified here (contig 16) is different from the one identified in the BW-BR library. This brings the number of known partial linkers cDNAs up to three out of the four known types of linker chains [22]. A sequence coding for a serine-threonine rich protein was also found and matches with a T-cell receptor protein sequence (contig 18, E-value = 0.58). Among the unknown sequences, the most abundant contig was composed of 24 sequences (contig 27).

**Table 4:** List of contigs with best E-values obtained on the TR-BW library sequences

Contig (TRbwC)	Number of sequences per contig	Putative identification on Blastx	E-value	GenBank accession number*
***Mitochondrial sequences***				

1	3	rRNA 16S large subunit 2542–2964	0	AY741662.1
2	2	rRNA 16S large subunit 2962–3499	0	AY741662.1
3	1	cytochrome c	4.10^-16^	EF648557
4	1	cytochrome c oxidase subunit I *Arabella*	3.10^-93^	EF648558
5	1	NADH dehydrogenase	5.10^-28^	EF648559

***All processes sequences (E-value<1)***				

6	5	carbonic anhydrase	3.10^-123^	AJ439711
7	1	carbonic anhydrase 3'UTR	3.10^-136^	AJ439711
8	19	hemoglobin (Hb) A1 chain (longer sequencing)	9.10^-20^	EF648449
9	28	small similar part of Hb A1 chain (2)	8.10^-6^	EF648560
10	1	small similar part of Hb A1 chain (3)	0.047	EF648450
11	7	small similar part of Hb A1 chain (4)	7.10^-3^	EF648451
12	1	new Hb A2 chain (hit with *Arenicola*)	9.10^-16^	EF648561
13	9	Hb B chain	8.10^-7^	P80592
14	1	new Hb B1 chain (hit with *Lamellibrachia*)	2.10^-11^	EF648568
15	2	Hb B2 chain	2.10^-12^	AAW78354
16	1	new extracellular Hb linker (hit with *Sabella*)	2.10^-8^	EF648562
17	5	myohemerythrin	2.10^-29^	EF648563
18	2	T-cell receptor	0.58	EF648564
19	2	small nuclear ribonucleoprotein	6.10^-22^	EF648565
20	2	oxidoreductase molybdopterin binding	0.32	EF648566
21	1	ubiquinol cytochrome reductase	2.10^-22^	EF648567
22	1	putative calmodulin	0.034	EF648452

***Hypothetical protein (E-value<1)***				

23	1	hypothetical protein	0.33	EF648569
24	1	hypothetical protein	0.46	EF648570
25	2	hypothetical protein *Oryza*	0.087	EF648571
26	1	hypothetical protein	0.19	EF648572

***Unknown sequences (E-value>1 or No Hits Found)***				

27	24	unknown sequence		EF648581
28 to 59	GenBank accession numbers EF648573 to EF648580, EF648582 to EF648601, EF648454 to EF648456, EF648453			

#### Subtracted body wall library (BW-TR)

Overall, the general results found for the BW-TR library (Table 5) are similar to those found for the BW-BR library (Table 3) although with a lower number of contigs. However, given the very large number of exoskeleton β-chitin-binding sequences (contig 3 and 4, comprising 24 and 59 sequences, respectively), we can suspect a less efficient normalization for these transcripts. The exoskeleton β-chitin-binding transcripts, galaxin, and myosin chains found in this library are the same as those found in the BW-BR library.

**Table 5:** List of contigs with best E-values obtained for the BW-TR library sequences

Contig (BWtrC)	Number of sequences per contig	Putative identification on Blastx	E-value	GenBank accession number*
***Mitochondrial sequences***				

1	2	rRNA 16S large subunit 2433–2964	0	AY741662.1
2	5	rRNA 16S large subunit 2962–3499	0	AY741662.1

***All processes sequences (E-value<1)***				

3	24	exoskeleton β-chitin-specific binding (3)	5.10^-61^	EU131642
4	59	exoskeleton β-chitin-specific binding (2)	5.10^-34^	EF648513
5	20	galaxin	6.10^-32^	EF648514
6	7	myosin regulatory light chain	1.10^-132^	AF173680
7	3	ficolin 2 precursor 108–211	7.10^-26^	EF648547
8	2	putative integrin isoform 2	0.33	EF648548
9	1	actin	6.10^-21^	EF648549
10	1	CCAAT-box DNA-binding	0.28	EF648550

***Unknown sequences (E-value>1 or No Hits Found)***				

11 to 17	GenBank accession numbers EF648546, EF648551 to EF648556			

### Checking the subtraction procedure and the specificity of the libraries

**Figure 2:**
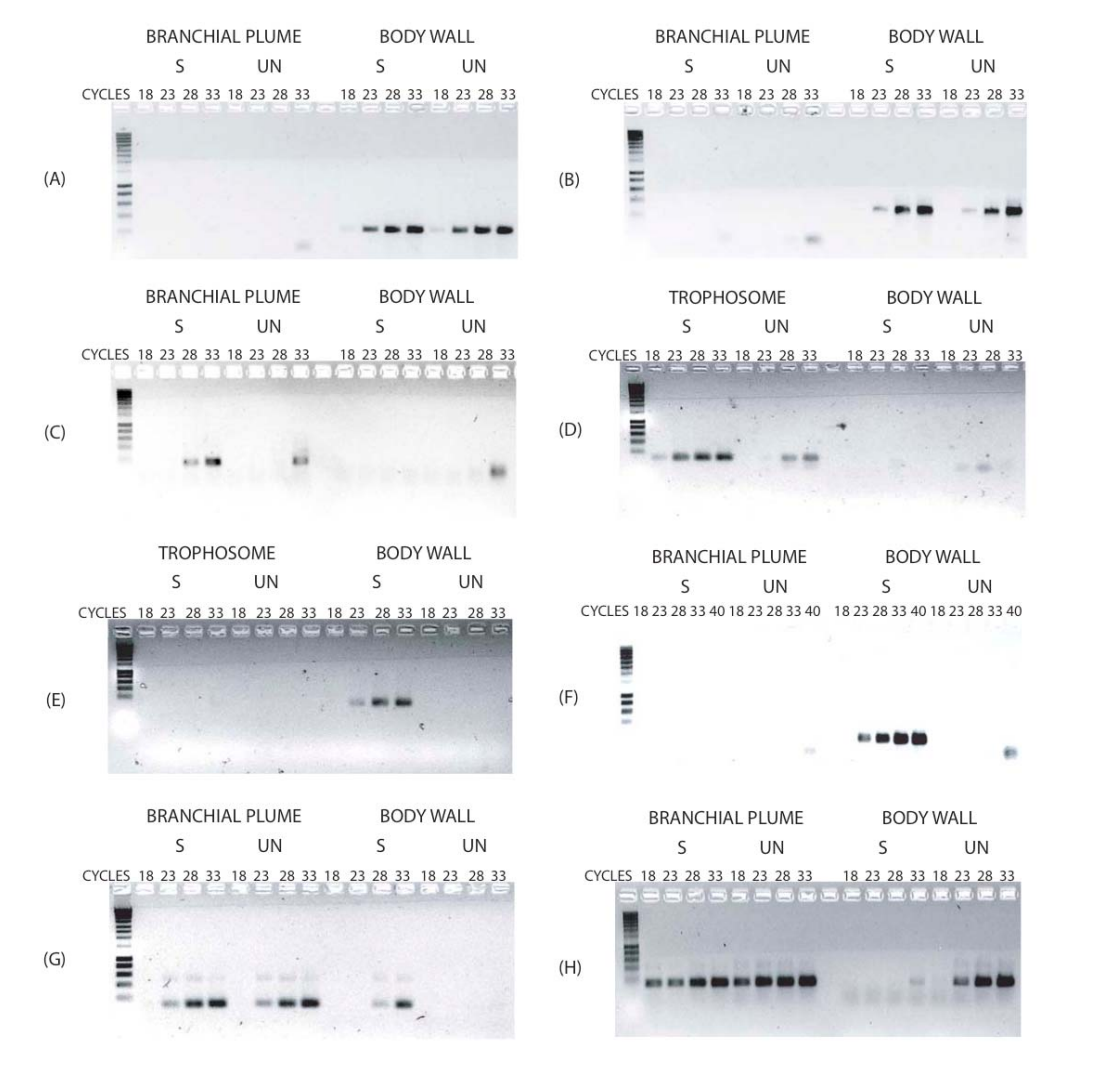
Typical PCR profiles obtained after amplification of fragments of interesting cDNAs. S = subtracted sample; UN = unsubtracted sample. (A) and (B) Abundant tissue-specific transcripts: exosqueleton β-chitin-binding transcript (A) and galaxin transcript (B). (C) and (D) Transcripts enriched after SSH procedure: chitinase precursor transcript (C) and RpCAtr (D). (E) and (F) Rare transcripts enriched after SSH procedure: RpCAbr transcript (E) and intracellular globin transcript (F). (G) Abundant transcript in one tissue and rare in other tissue: MVP transcript. (H) Non equally subtracted transcript: cytochrome c oxidase subunit I transcript. The faint bands appearing at a smaller size than expected in some wells are interpreted as non-specific amplification (possible primer dimerization under specific conditions).

We only found 3 common cDNAs between the BR-BW and BW-BR libraries and 2 common cDNAs between the TR-BW and BW-TR ones. We were able to assess the degree of successful subtraction for several transcripts by performing regular PCR on subtracted and unsubtracted poly-A cDNA pools. Some results are shown in Fig. 2.

The profiles of abundant tissue-specific transcripts are presented in Fig. 2A (exoskeleton β-chitin-binding transcript) and 2B (galaxin transcript). The exoskeleton β-chitin-binding transcript was clearly present in the body wall tissue (Fig. 2A) in both unsubtracted cDNA sample and subtracted BW-BR cDNA sample (before and after SSH procedure respectively). The same profile was obtained for the galaxin transcript (Fig. 2B).

Typical profiles of enrichment of transcripts after SSH procedure are shown in Fig. 2C,D. The chitinase precursor sequence is enriched by the SSH, appearing on agarose gel after 28 cycles instead of 33 cycles on the branchial plume cDNA (Fig. 2C) and RpCAtr sequence amplification is visible after 18 cycles instead of 28 cycles from the trophosome cDNA before the SSH procedure (Fig. 2D).

Fig. 2E and 2F show typical profiles of amplification of rare transcripts. RpCAbr amplification can be seen on the BW-TR cDNA pool after 23 cycles and not from the body wall unsubtracted cDNA, even at 33 cycles (Fig. 2E). Amplification of the intracellular globin sequence can be seen on the BW-BR cDNA pool after 23 cycles and not from the body wall unsubtracted cDNA, even after 40 cycles (Fig. 2F).

Fig. 2G shows results of the amplification profile of MVP. This transcript appeared abundant in one tissue (the branchial plume) and rare in another (the body wall). Only the subtraction procedure allowed its detection in the body wall (not detected in the unsubtracted sample, even after 33 cycles).

Finally, Fig. 2H illustrates the difference of SSH efficiency of a same transcript in two different subtraction procedures (BR-BW and BW-BR). A successful amplification of cytochrome c oxidase I (ccox I) was obtained in both branchial plume and body wall unsubtracted cDNA pools after 18 and 23 cycles respectively. No amplification was observed in the BW-BR subtracted cDNA pool, in contrast with the BR-BW one. It seems that the subtraction was successful in the BW-BR library whereas this transcript could not be successfully subtracted in the BR-BW library after SSH procedure.

### Relative expression levels of some target genes over the three types of tissues

We used quantitative PCR on some transcripts to further assess tissue specificity and gain data on the relative level of expression in each tissue starting from total cDNA. For all studied transcripts, PCR were performed from different initial amount of total cDNA in order to construct standard curves. The equations of the curves are reported in Table 6. All PCR efficiencies (E in Table 6), calculated based on the slopes of the curves, varied between 92 and 107%. For the dilution range we chose, we could only amplify the Major Vault Protein (MVP) transcript in the branchial plume and the body wall tissues, the chitinase precursor (ChPr) in the branchial plume tissue, and the myohemerythrin (MH), T-cell receptor (TCR) and contig 27 from TR-BW library (TRbwC27, unknown protein) transcripts in the trophosome tissue.

**Table 6:** Equations of the standard curves obtained by amplification from total cDNA samples of branchial plume, trophosome and body wall tissues

Standard curve equation and efficiency (E) in the different tissues (calculated from one sample each time						
Transcript	Branchial plume		Trophosome		Body wall	

18S	y = -3.31x + 24.67	E = 101 %	y = -3.38x + 28.30	E = 98 %	y = -3.29x + 24.55	E = 101 %
RpCAbr	y = -3.24x + 39.13	E = 103 %	y = -3.37x + 51.40	E = 98 %	y = -3.30x + 44.66	E = 101 %
RpCAtr	y = -3.30x + 49.19	E = 101 %	y = -3.21x + 38.84	E = 105 %	y = -3.25x + 47.08	E = 103 %
MVP	y = -3.35x + 39.63	E = 99 %	nd		y = -3.24x + 42.51	E = 104 %
Cathep	y = -3.22x + 43.13	E = 104 %	y = -3.33x + 47.41	E = 100 %	y = -3.26x + 43.98	E = 102 %
ChPr	y = -3.37x + 39.57	E = 98 %	nd		nd	
16S	y = -3.31x + 29.86	E = 101 %	y = -3.17x + 38.77	E = 107 %	y = -3.43x + 34.80	E = 96 %
ccoxI	y = -3.54x + 36.05	E = 92 %	y = -3.42x + 40.74	E = 96 %	y = -3.46x + 38.51	E = 94 %
ATPF1	y = -3.43x + 41.54	E = 96 %	y = -3.41x + 44.95	E = 96 %	y = -3.34x + 43.14	E = 99 %
MH	nd		y = -3.24x + 41.08	E = 103 %	nd	
TCR	nd		y = -3.27x + 41.34	E = 102 %	nd	
TRbwC27	nd		y = -3.36x + 39.07	E = 98 %	nd	

nd: not detected

For each sequential dilution, PCR reactions were performed in triplicates. PCR efficiencies calculated from the slope of each curve are given.

**Figure 3:**
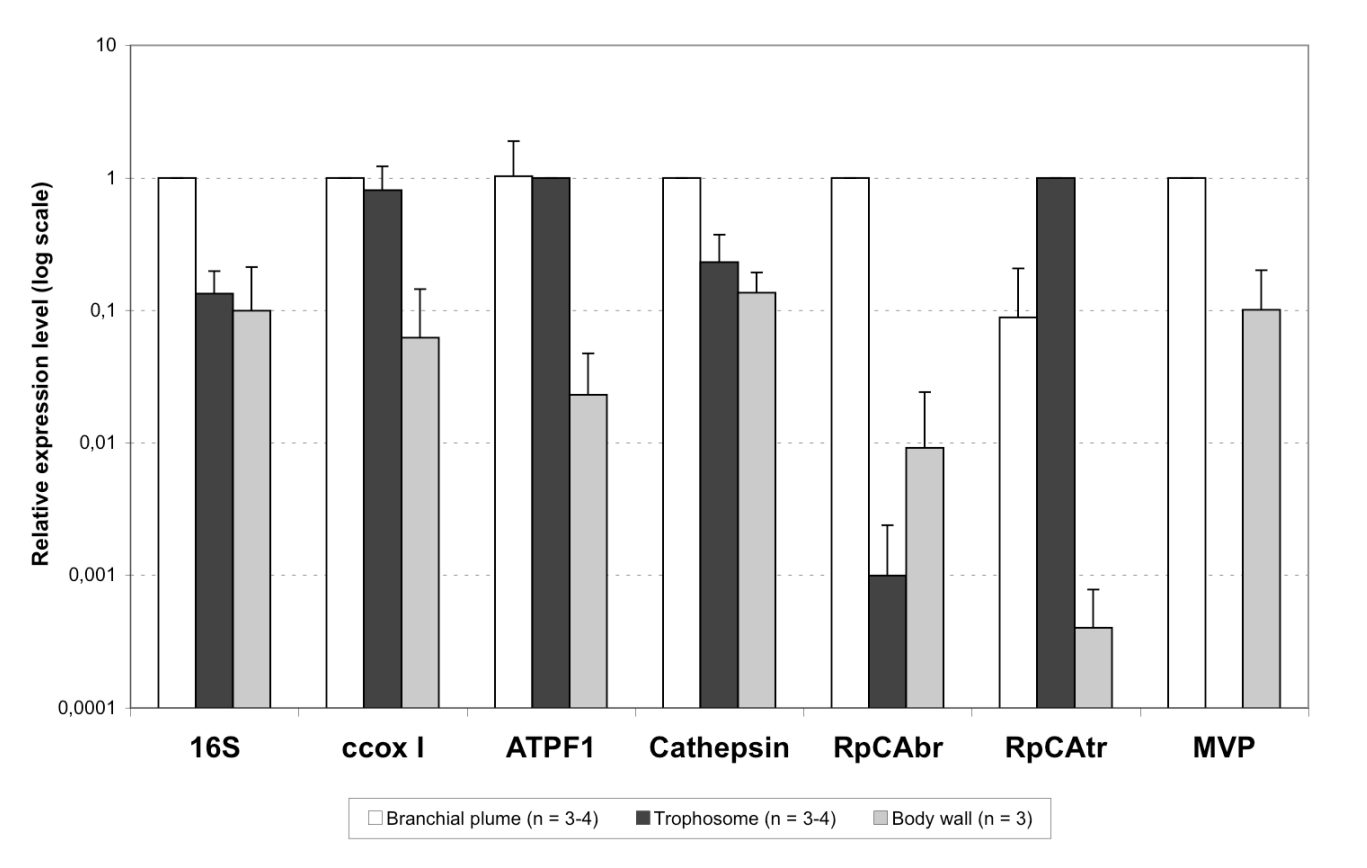
Relative expression levels of ribosomal RNA 16S, ccox I, ATPF1, Cathepsin, RpCAbr, RpCAtr, and MVP transcripts in the branchial plume, trophosome and body wall tissues. For each transcript, the calibrator tissue was chosen as the tissue with the higher expression: the branchial plume was the calibrator for ribosomal RNA 16S, ccox I, Cathepsin, RpCAbr and MVP amplifications, the trophosome was the calibrator for RpCAtr and ATPF1 amplifications. The number of tissue replicates (n) ranges from 3 to 4, and corresponds to the number of intra-individual tissue pairs we had.

Relative expression levels were calculated between the different tissues of a whole organism after normalization of the transcripts amplifications with the 18S reference gene. The results from the analysis of several individuals are shown in Fig. 3. The 16S ribosomal gene has 7.5-fold and 10-fold higher expression levels in the branchial plume compared to the trophosome and the body wall, respectively. The ccox I and ATP synthase F1 transcripts were equally present in the branchial plume and trophosome tissues but comparatively less abundant in the body wall tissue (about 16-fold and 43-fold, respectively). The new CA sequence (RpCAbr) is preferentially expressed in the branchial plume tissue compared to the trophosome (1,000-fold less expression) and the body wall (109-fold less expression) tissues. On the opposite, the RpCAtr transcript was more abundant in the trophosome tissue than in the branchial plume (12-fold less expression) and the body wall (2,500-fold less expression) tissues. Relative quantification analyses also showed that cathepsine L-like genes are also up-regulated in the branchial plume tissue compared to the trophosome (about 4-fold less expressed) and the body wall (about 7-fold less expressed) tissues of the worm. The MVP transcript was nearly 10-fold more abundant in the branchial plume than in the body wall tissue but we could not detect it in the trophosome tissue total cDNA.

Relative expression calculations for ChPr, MH, TCR and TRbwC27 sequences could not be made because we could only generate standard curves from the branchial plume total cDNA (for ChPr transcript) or from the trophosome total cDNA (for MH, TCR and TRbwC27) which indicate that these transcripts are tissue-specific.

## Discussion

### Use of the SSH method for the study of symbiosis

We used SSH on different tissues from a single individual to look for genes involved in the functioning of the symbiosis because it is not possible to obtain aposymbiotic adult *Riftia pachyptila*. The body wall was used as a reference tissue to find specific proteins expressed in the gills (main metabolite exchange organ with the milieu) and in the trophosome (organ that houses the symbiotic bacteria). We then focused our attention on some chosen transcripts for a quantitative analysis. The remaining unidentified sequences (many of which could correspond to 3'UTR portions of cDNA) could prove interesting. Their future identification will require either a RACE approach or hybridization on a full length cDNA library.

### Efficiency of the SSH method

All the 10 sequences that were more closely studied by quantitative PCR showed differential expression in agreement with the subtractive libraries where they were found. A transcript obtained in a given library showed the highest expression in the expected tissue, as evidenced by checking the result with rapid PCR validation (Fig. 2) and from several individuals by quantitative PCR (Fig. 3). In addition, although constitutively expressed in all cells of all tissues, only one tubulin transcript sequence was obtained from the BR-BW library, and one actin sequence from the BW-TR library. This demonstrates the adequate subtraction of these common sequences. However, some sequences are sometimes highly represented, possibly indicating a subtraction that was not as efficient. ccox I, for example, could not be eliminated in the BR-BW cDNA pool, but this may be due to the fact that it was more expressed in the branchial plume than in the body wall tissue. Strangely however, we recovered the RpCAbr transcript from the BW-BR library (Table 3) although it was 109-fold less abundant in the body wall than in the branchial plume tissues (Fig. 3).

As noticed by Ji and coworkers [23], SSH PCR favors highly differentially expressed genes. From our quantitative PCR results, some transcripts showed such high differential expression (e.g. RpCAbr in the branchial plume compared to the other tissues, and RpCAtr in the trophosome compared to the other tissues). These authors suggest that the primary application of SSH PCR should be the detection of dramatic alteration of gene expression, as it is for example the case for gene expression profiling of two different tissues. Our use of SSH for comparing pairs of tissues seems very appropriate.

### Proteins degradation and turnover in the branchial plume tissue

Some transcripts were preferentially expressed in the branchial plume tissue. Relative quantification of the cathepsin transcript (a degradation enzyme found in lysosomes) revealed a more important expression in the branchial plume tissue, compared to the body wall (protected by the tube) and the trophosome. The plume is the only organ in direct contact with sea-water, and thereby strongly exposed to hydrogen sulfide and other toxic molecules such as heavy metals which are abundant in the hydrothermal vent environment. Electron-dense organelles (EDO) seem to be very common in tissues of sulfide-adapted marine annelids. Such structures have previously been observed in both the *Riftia pachyptila *epidermal body wall [24] and the branchial plume organ (Ann Andersen, personal communication). Arp and coworkers [25] hypothesized that EDO structures could actually be secondary lysosomes. This could be in agreement with our results of cathepsin expression, found to be highest in this highly exposed tissue. Julian and coworkers [26] showed that even in sulfide-tolerant organisms like the annelid *Glycera dibranchiata*, sulfide exposure poisons the mitochondria, leading to depolarization that is not reversible. Lysosomes could degrade mitochondria that have been damaged by sulfide exposure. Besides, we also found a Rab5 GDP/GTP exchange factor (BRbwC22), which is a member of the Ras superfamily of GTPases. This protein, involved in vesicle trafficking, is known to be located in early endosomes that precede the formation of lysosomes.

Interestingly, another pathway of degradation could also be involved since we obtained a Valosin-Containing Protein (VCP, BRbwC21), which is required in ubiquitin-proteasome degradation [27]. The existence of two VCP transcripts has recently been demonstrated in an annelid, the earthworm *Eisenia fetida *[28]. Our sequence shows the best homology scores with the predicted VCP protein sequence of *Strongylocentrotus purpuratus *[Expect = 0.014] and the eVCP-1 isoform from *Eisenia fetida *[Expect = 0.025] which is ubiquitously expressed in this worm [28].

If EDOs do represent autophagic degradation of organelles as suggested by Arp and coworkers [25], rapid replacement of organelles should take place [29]. The high expression level of ribosomal 16S, an essential gene for the translation of mitochondrial messenger RNAs into proteins, and the presence of some transcripts linked to transcription (BRbwC19 and 20, Table 2) is then consistent with a high protein turnover in this tissue.

Sulfide oxidation with the concomitant production of ATP by the mitochondria of the annelid *Arenicola marina *has been shown [30]. However, no protein sequence was found here that would suggest a similar property of *R. pachyptila *mitochondria.

### Hydroxylamine reductase protein

Formerly known as prismane, the hydroxylamine reductase is a member of the Hybrid-Cluster Protein (HCP) family and is thought to play a role in nitrogen metabolism. It catalyses the reduction of hydroxylamine to form ammonia using NADH. In rat liver mitochondria, this enzyme is firmly attached to the mitochondrial membrane [31] and its activity can prevent hydroxylamine to inhibit mitochondrial respiration [32]. However, blasts of our sequence did not match with the few eukaryotic sequences available but resulted in 100 bacteria sequences hits. The best ones are those of the Actinobacteria *Salinispora arenicola *[Expect = 5e-19] and the α-proteobacteria *Rhodospirillum rubrum *[Expect = 5e-19]. Because this sequence did not match any sequence in the *Riftia *symbiont genomic database, it could be contamination from bacteria living close to the branchial plume of the worm.

### Major Vault Protein gene expression

The Major Vault Protein (MVP) (100 kDa) is the major protein component of vaults, ribonucleic particles of 13MDa. Some studies established that vaults could be involved in nucleocytoplasmic transport of ribosomes and/or mRNA [33]. This could be coherent with our results of 16S expression obtained on the branchial plume tissue in which probable high transcription levels of this protein occur. Other studies indicate the participation of MVP in drug resistance mechanisms where it could act as a nucleocytoplasmic and vesicular transporter of drugs and/or metabolites to transport them to exocytotic vesicles or proton pumps [34,35]. It could be evidenced that MVP gene in *Mytilus edulis *was predominantly expressed in epithelia-rich tissues such as the gills and digestive gland and could be involved in multixenobiotic resistance [36]. In our study, MVP transcript is preferentially expressed in the branchial plume tissue compared to the body wall, while no MVP transcript was detected in the trophosome samples. The presence of such a protein in the branchial plume tissue may be used to temporarily immobilize toxic molecules before they are processed.

### Chitinase gene expression

Interestingly, a chitinase precursor was recovered as a branchial plume specific transcript. A previous report indicated chitinase activity in the opisthosome and branchial plume of *R. pachyptila *[37]. Chitin is a major component of the tube of *R. pachyptila*, produced by specialized glands located in the body wall and the vestimentum [38]. Chitinase activity was suggested to be involved in tube growth and tube shape modifications [37]. A chitinase sequence was recently discovered in the hydroid cnidarian *Hydractinia *[39] and a possible role of chitinase enzyme in pattern formation and allorecognition was suggested. Interestingly, the transcript was exclusively expressed in ectodermal tissues of the animal, and the authors also suggested a possible role in host defense against pathogens. Such a hypothesis could be interesting to explore given our quantitative PCR experiments because we only could amplify this transcript from cDNA from the branchial plume, the only organ in contact with the environmental sea water.

### Tissue-specific expression of different carbonic anhydrases

Our quantification analyses showed a higher abundance of the RpCAbr transcript in the branchial plume compared to the trophosome (present at very low levels) and the body wall tissues. In contrast, the RpCAtr transcript was very abundant in the trophosome compared to the branchial plume (medium levels) and the body wall tissues. Fluorescent *In Situ *Hybridization confirmed the co-expression of the two transcripts in the branchial plume in contrast with the trophosome where only one transcript could be detected [19]. An alignment of these translated CA cDNAs with vertebrate and non-vertebrate CA protein sequences revealed the conservation of most amino acids involved in the catalytic site, indicating that the two proteins are probably functional if the cDNAs are translated [19].

### Myohemerythrin, T-cell receptor, and unidentified transcripts from the trophosome library

A complete coding sequence obtained from the TR-BW library (contig 17, Table 4) showed a very high homology score with a myohemerythrin sequence from the Sipuncula *Sipunculus nudus *[GenBank:CAG14944] (Expect = 1e-11). The complete *Riftia *sequence has an open-reading frame of 120 amino acids. Myohemerythrin is an oxygen-binding protein that participates in the storage of oxygen in muscles. Such a protein could be involved in the regulation of cadmium levels in the gut of the annelid *Nereis diversicolor *[40]. In *Hirudo medicinalis*, it would have indirect antibacterial properties by regulating free iron availability to deprive bacteria of iron essential for their growth [41].

Both TCR and TRbwC27 cDNAs showed specific expression in the trophosome tissue where they could be essential. The TCR transcript first caught our attention because it matched a sequence fragment coding for a T-cell receptor, which is a complex of integral membrane proteins that participates in the activation of T-cells in response to the presentation of an antigen. The trophosome is mostly composed of bacteriocytes which house bacterial cells in intracellular vacuoles and cellular recognition may be very important for the functioning of this tissue. As for the TRbwC27 it is a large fragment of 273 nucleotides which is highly represented in our subtracted library but did not reveal reliable Blastx homology E-values.

## Methods

### Animals and sampling

Specimens of *Riftia pachyptila *were collected at the Oasis site (17°25.385 S, 113°12.280 W) at 2600 meters-depth along the South East Pacific Rise during the BIOSPEEDO 2004 cruise. For each individual, parts of the branchial plume, trophosome and body wall tissues were isolated on ice, placed in RNAlater (Ambion) for 24 h at 4°C and then frozen in liquid nitrogen.

### RNA extraction

Plume, trophosome and body wall tissue samples were ground individually in liquid nitrogen under RNase-free conditions. For each tissue, total RNA was extracted using the RNAble buffer (Eurobio) following the manufacturer's instructions. Then, both for library constructions and for complete sequencing, messenger poly-A RNAs were purified using the oligo-dT resin column of the mRNA Purification Kit (Amersham).

### Construction of subtractive tissue-specific cDNA libraries

Libraries were constructed from tissues taken from a single individual, thereby representing a single organism's transcriptome. A total of 4 libraries were produced: branchial plume vs. body wall subtracted library (and its reciprocal) and trophosome vs. body wall subtracted library (and its reciprocal). For all tissue pairs, cDNA synthesis as well as subtractive suppressive hybridization (SSH, [[42], [43]]) including steps of adaptor ligation, subtractive hybridization, and selective amplification were performed following the protocol of the Clontech PCR-Select™ cDNA Subtraction Kit (BD Biosciences).

In the BR-BW library, SSH was performed to produce a cDNA library enriched in branchial plume specific transcripts. The tester sample was the cDNA population from the branchial filaments (BR) and the driver sample was the cDNA population from the body wall tissue (BW). In the BW/BR library the tester and driver samples were reversed, and SSH was performed to produce a cDNA library enriched in body wall specific transcripts. In the TR-BW library, the tester sample was the cDNA population from the trophosome (TR) and the driver sample was the cDNA population from the body wall tissue (BW). In the BW-TR library, the tester and driver samples were reversed.

### Cloning and sequencing

For each SSH procedure, the whole amplification product was cloned into the TOPO^®^-TA cloning vector (Invitrogen), producing a range of cDNA fragment sizes. Nearly 200 cDNA fragments were sequenced for each library. Plasmid DNA from individual colonies were purified with the FlexiPrep kit (Amersham) and used in a dye-primer cycle sequencing reaction with T3 or T7 universal primers and the Big Dye^® ^Terminator V3.1 Cycle Sequencing kit (Applied Biosystems). Reactions were then run on a 16-capillary 3130 Applied Biosystems sequencer.

### Sequence analysis and homology search

Most of chromatograms obtained after sequencing were treated with PHRED [ [44]] and Seqclean software (TGIR, the Institute for Genomic Research, Rockville, MD, USA) to remove vector and adaptors sequences. Progressively, additional sequences were treated manually. Clustering was performed with the TGICL programs, Megablast and CAP3 [[45]]. Clusters and contigs were formed on the whole set of sequences and also individually for each of the four libraries. Contigs were then verified manually to detect possible chimeras. BLAST analyses of the cDNA libraries sequences were performed on the NCBI server. The assembled sequences were analyzed for homology with known sequences in databases using the BlastX and BlastN programs [[46]] and also treated with the PhyloGena software [[47]] which combines both homology searching and phylogenetic reconstruction to verify the homology attributions.

The redundancy corresponds to the probability that a newly sequenced cDNA was previously obtained. Redundancy rate was calculated with the formula:

R = (1- (Nu/Nt)) × 100, where Nu is the number of unique sequences and Nt is the total number of sequences.

### Validation of differential expression by simple PCR

Unsubtracted and subtracted PCR samples (respectively before and after SSH procedure) were obtained as recommended in the Clontech PCR-Select™ cDNA Subtraction Kit (BD Biosciences). These samples were then diluted ten-fold in sterile milliQ water. For each transcript tested, PCRs were conducted on these diluted unsubtracted and subtracted samples with specific forward and reverse primers (Table 7). Each reaction mixture was composed of 1 μl of cDNA sample; 1.2 μl of specific forward primer (10 μM), 1.2 μl of specific reverse primer (10 μM), 22.4 μl of sterile water, 3 μl of 10X PCR reaction buffer, 0.6 μl of dNTP Mix (10 mM) and 0.6 μl of 50X Advantage cDNA Polymerase Mix (BD Biosciences). The following thermal cycling program was used for 33 cycles: 94°C for 30 s, 60°C for 30 s, and 68°C for 2 min. For each cDNA pool tested, a 5 μl-aliquot was removed from the reaction mixture every 5 cycles, starting at the end of cycle 18.

**Table 7:** Primers sequences used for the transcripts amplifications

**Amplification of transcripts by classical PCR**	
Primers	Sequence

ExosqF	5' TGC AGG CGA TGC GAG TGC 3'
ExosqR	5' GCT ACA ACA GCG GTT AGG 3'
GalaxinF	5' ATT TCG TTT GCA ACA GCC 3'
GalaxinR	5' CTT CCT CTG CAG CAC TGG 3'
ChPrF	5' AAT TCT GAG ACC GGT GAC C 3'
ChPrR	5' TCC AAG ACC GTG TTG AGC 3'
RpCAtrF	5' TAC AAA GAT CCA ATC CAG C 3'
RpCAtrR	5' ACG AGG ACG ACA CCT GG 3'
RpCAbrF	5' TAC AAG GAT GCC ATT AGC 3'
RpCAbrR	5' AGA GCA GCA GAC CTT ACG 3'
IntraGlobF	5' GGA AAG GAC GTC GAC AGC 3'
IntraGlobR	5' TGC TGC TTG GTT AGT CCC 3'
MVPF	5' GAG AAC AGA ATG ACA TGG 3'
MVPR	5' TTT CAC CTG CAT CTC GGG 3'
ccoxIF	5' ACA GGT TTA GTA GCC ACT 3'
ccoxIR	5' GTG TTG ATA TAG GAC TGG 3'

**Amplification of transcripts by quantitative PCR**	

Primers*	Sequence

RpCAbrFq	5' TGG TTT CAC CCC GTC GAA 3'
RpCAbrRq	5' GGT CTG GTC TTT TCT CGC CAT A 3'
RpCAtrFq	5' GCC AGG TGT CGT CCT CGT T 3'
RpCAtrRq	5' TCA CAA ATG TCC AGT GCC AGT T 3'
16SFq	5' CGT AAG ACT ATA GCT GGT TTT CCA AA 3'
16SRq	5' TTA TCA AAG ATT TTT TCT TGG TTC ACT AAT 3'
MVPFq	5' GAT TGA GAC AAC CAA GTT CAG GAA 3'
MVPRq	5' CTG GCG ATT GCC TGA ATT G 3'
CathepFq	5' TAC ATG GCC CGT AAT AAG GAC A 3'
CathepRq	5' GCT GGC TTG TGA TGC AAC AC 3'
ccoxIFq	5' CTA ATG GGA GGC TTC GGA AAC 3'
ccoxIRq	5' AGG TGC CCC TAG CAT TAA AGG 3'
ATPF1Fq	5' TGC AGG ACA TCA TTG CCA TC 3'
ATPF1Rq	5' TGT CCT CCT GGG ACA ACT CG 3'
ChPrFq	5' GTC GTC GGA ATG GCG AGT TA 3'
ChPrRq	5' AGC GTT GCT GGC TGT TTT G 3'
MHFq	5' AGA GGC ACA CCA ACA ACC GT 3'
MHRq	5' CCC GAT TGG TTC ATC ACA GC 3'
TCRFq	5' AAT CCG ACG TGG CGA TCA T 3'
TCRFq	5' GGT CAT TGT TGT TGC CTG GG 3'
TRbwC27Fq	5' CGA CGG TGG TAC CCC GTA TA 3'
TRbwC27Rq	5' CCG CAA CCT TTG AAC CTC AG 3'

*Primers designed by Primer Express software (ABI PRISM™)

### SYBR Green quantitative PCR

#### Reverse transcription (RT)

Fresh RT reaction was carried out with a random primer on each total RNA sample (branchial plume, trophosome and body wall). The reaction mixture was composed of 2 μl of M-MLV 5X RT buffer; 0.5 μl of BSA (10 mg/ml), 1 μl of total RNA (1.24 μg/μl), 2.5 μl of dNTP (4 mM total), 1.5 μl of Random Primer 9 (Ozyme) (100 ng/μl), 3 μl DEPC water. The reaction mixtures were then incubated at 80°C for 5 minutes and placed on ice. M-MLV RT was added (1 μl) to each reaction mixture and all reactions were incubated at 42°C for 1 hour and finally placed on ice.

#### Amplification

Specific pairs of primers for some target genes (Table 7) were designed using the software Primer Express. 18S rRNA transcript was chosen as a reference gene for the normalization of expression data and was amplified with the 18h and 18L primers designed by Halanych and coworkers [ [48]]. For amplifications, the Power SYBR Green PCR master mix (PE Applied Biosystems) was used in 23 μl reaction mixtures on a Chromo4™ System CFB-3240 (BIORAD). Amplification conditions were 40 cycles with the following profile: 95°C for 30 s, 60°C for 30 s, and 72°C for 1 min.

#### Standard curves

In order to estimate the relative expression levels of each transcript by the 2^-ΔΔCt ^method, we calculated PCR efficiencies of the transcripts amplifications to verify they do not highly differ from the reference transcript (rRNA 18S) amplification. PCR were performed from a dilution range of total cDNA from each tissue (from one sample). Therefore, we performed PCR starting from total cDNA amounts ranging from 6.2 pg to 620 ng. Each PCR reaction was performed in triplicate. For each initial template quantity, we looked at the threshold cycle, the Ct, corresponding to the number of cycles required to reach a set quantity of amplified cDNA during the exponential phase. The standard curves were generated by plotting the log of the initial template concentration against the Ct generated for each dilution.

#### Data analysis

For each transcript, the efficiency (E) was calculated from the slope (S) of the standard curve using the formula:

E = 10^-1/S ^- 1

Once differences between efficiencies of reference gene and target gene amplifications were approximately equal, we calculated the relative expression level for each gene analyzed. In each tissue, amplification of the target transcript was compared to the endogenous control amplification in order to get the normalized number of cycles (NNC):

NNC = Ct target - Ct 18S

Then, for relative quantification measurement, we used the 2^-ΔΔCt ^method [ [49]] for individuals for which we had analyzed at least one pair of tissues. Relative quantification results were obtained by comparing levels of expression with the calibrator tissue, the latter being chosen as the tissue for which the better expression was observed with the following calculation:

Relative expression level = 2^-(NNC^_sample_^-NNC^_calibrator_).

## Competing interests

The author(s) declares that there are no competing interests.

## Authors' contributions

SS carried out the experimental work, the sequence analysis and drafted the manuscript. SH participated in sample acquisition, sequence analysis and helped to draft the manuscript. FHL conceived the study, participated in sample acquisition and coordination, and helped to draft the manuscript. All authors read and approved the final manuscript.
